# A Natural Variant of the T Cell Receptor-Signaling Molecule Vav1 Reduces Both Effector T Cell Functions and Susceptibility to Neuroinflammation

**DOI:** 10.1371/journal.pgen.1006185

**Published:** 2016-07-20

**Authors:** Sahar Kassem, Guillaume Gaud, Isabelle Bernard, Mehdi Benamar, Anne S. Dejean, Roland Liblau, Gilbert J. Fournié, Céline Colacios, Bernard Malissen, Abdelhadi Saoudi

**Affiliations:** 1 UMR Inserm, U1043, Toulouse, France; 2 UMR CNRS, U5282, Toulouse, France; 3 Université de Toulouse, UPS, Centre de Physiopathologie de Toulouse Purpan, Toulouse, France; 4 Centre d’Immunologie de Marseille-Luminy, Aix Marseille Université UM2, Inserm, U1104, CNRS UMR7280, Marseille, France; 5 Centre d’Immunophénomique, Aix Marseille Université UM2, Inserm US012, CNRS UMS3367, 13288 Marseille, France; The Jackson Laboratory, UNITED STATES

## Abstract

The guanine nucleotide exchange factor Vav1 is essential for transducing T cell antigen receptor signals and therefore plays an important role in T cell development and activation. Our previous genetic studies identified a locus on rat chromosome 9 that controls the susceptibility to neuroinflammation and contains a non-synonymous polymorphism in the major candidate gene *Vav1*. To formally demonstrate the causal implication of this polymorphism, we generated a knock-in mouse bearing this polymorphism (Vav1^R63W^). Using this model, we show that Vav1^R63W^ mice display reduced susceptibility to experimental autoimmune encephalomyelitis (EAE) induced by MOG_35-55_ peptide immunization. This is associated with a lower production of effector cytokines (IFN-γ, IL-17 and GM-CSF) by autoreactive CD4 T cells. Despite increased proportion of Foxp3+ regulatory T cells in Vav1^R63W^ mice, we show that this lowered cytokine production is intrinsic to effector CD4 T cells and that Treg depletion has no impact on EAE development. Finally, we provide a mechanism for the above phenotype by showing that the Vav1^R63W^ variant has normal enzymatic activity but reduced adaptor functions. Together, these data highlight the importance of Vav1 adaptor functions in the production of inflammatory cytokines by effector T cells and in the susceptibility to neuroinflammation.

## Author Summary

The understanding of the physiological role of Vav1, a key regulator of T cell receptor signaling, was primarily inferred from studies using Vav1-deficient mice. Such models, however, provide little insight on how polymorphisms leading to quantitative changes in Vav1 activity could affect immune system functions. In the present study, we focused on a recently identified Vav1^R63W^ natural variant that has been supposed to play a central role in the susceptibility to neuroinflammation. Using a Vav1^R63W^ knock-in mouse model, we show that Vav1^R63W^ leads to defects in adaptor functions and reduces the susceptibility to experimental autoimmune encephalomyelitis, together with an intrinsic defect in the production of Th1/Th17 cytokines by autoreactive effector CD4 T cells. Thus, our study highlights the importance of Vav1 adaptor functions in CD4 T cells differentiation and suggests that genetic or acquired alterations of this Vav1 function could play a major role in susceptibility to Th1/Th17 mediated diseases.

## Introduction

The guanine nucleotide exchange factor (GEF) Vav1 is essential for transducing T cell antigen receptor (TCR) signals and therefore plays a critical role in the development and activation of T cells [1–5]. Following TCR engagement, Vav1 becomes rapidly tyrosine phosphorylated by kinases of the Src and/or Syk family. This phosphorylation relieves Vav1catalytic Dbl homology (DH) domain and causes Vav1 to promote GDP-GTP exchange on the Rho, Rac1 and Cdc42 small GTPases [6, 7]. In addition to this GEF activity, the CH, SH2 and SH3 domains of Vav1 allow its incorporation into the LAT-Grb2-Gads-PLCγ1-SLP76 micro-clusters that form at the immunological synapse. Binding of Vav1 appears to stabilize this molecular complex thereby controlling PLCγ and nuclear factor of activated T cells (NFAT) activation [1, 8, 9]. In Vav1-deficient mice, T cell development is partially blocked at the pre-TCR checkpoint in the thymus and both positive and negative selections of T cells are strongly impaired [3, 5]. Furthermore, TCR-induced activation and proliferation is greatly diminished in Vav1-deficient T cells, due to reduced TCR-induced signaling that impacts on Ca^2+^ flux, on the activation of Erk, protein kinase D1 (PKD1) and serine-threonine kinase Akt, and on the activity of transcription factors such as NFAT and nuclear factor κB (NF-κB) [1, 2, 10]. A recent study has shown that many critical events involved in T cell activation are mediated by either the GEF or the scaffolding activities of Vav1 [11]. The GEF activity of Vav1 is necessary for T cell development and for the optimal activation of T cells, including signal transduction to Rac1, Akt, and integrins. In contrast, Vav1 GEF activity is not required for TCR-induced Ca^2+^ flux, activation of Erk and PKD1, cell polarization and development of regulatory T cells (Treg).

Lewis (LEW) and Brown-Norway (BN) rats behave in opposite ways concerning their susceptibility to autoimmunity, allergy and infectious diseases [12, 13]. Genetic dissection using these rat strains has identified a locus of 117 kb on chromosome 9 that controls natural Treg development [14]. Fine mapping of this locus revealed a non-synonymous SNP in the *Vav1* gene leading to the substitution of an arginine residue by a tryptophan at position 63 (R63W) in BN rats. Interestingly, this 117 Kb interval is fully included in the *Eae4* locus of 1 cM that controls the susceptibility to central nervous system (CNS) inflammation [15]. Although this study suggested that Vav1 could be involved, one important limitation was the possibility that other genetic variants contained in the 117 Kb fragment besides the Vav1^R63W^ polymorphism could be responsible for these phenotypes.

Here, we sought to unequivocally test the involvement of the Vav1^R63W^ polymorphism in the susceptibility to CNS inflammation and to determine its mechanisms of action. To this aim, we generated a knock-in mouse model in which the arginine at position 63 was replaced by a tryptophan residue. Using this model, we show that Vav1^R63W^ mice display reduced susceptibility to experimental autoimmune encephalomyelitis associated with a lower production of effector cytokines by autoreactive CD4 T cells that is intrinsic to effector CD4 T cells. Finally, we provide a mechanism for the above phenotype by showing that the Vav1^R63W^ variant has normal enzymatic activity but reduced adaptor functions. Together, these data highlight the importance of Vav1 adaptor functions in the production of inflammatory cytokines by CD4 T cells and in the susceptibility to neuroinflammation.

## Results

### The Vav1^R63W^ polymorphism impairs thymic selection

**Fig 1:**
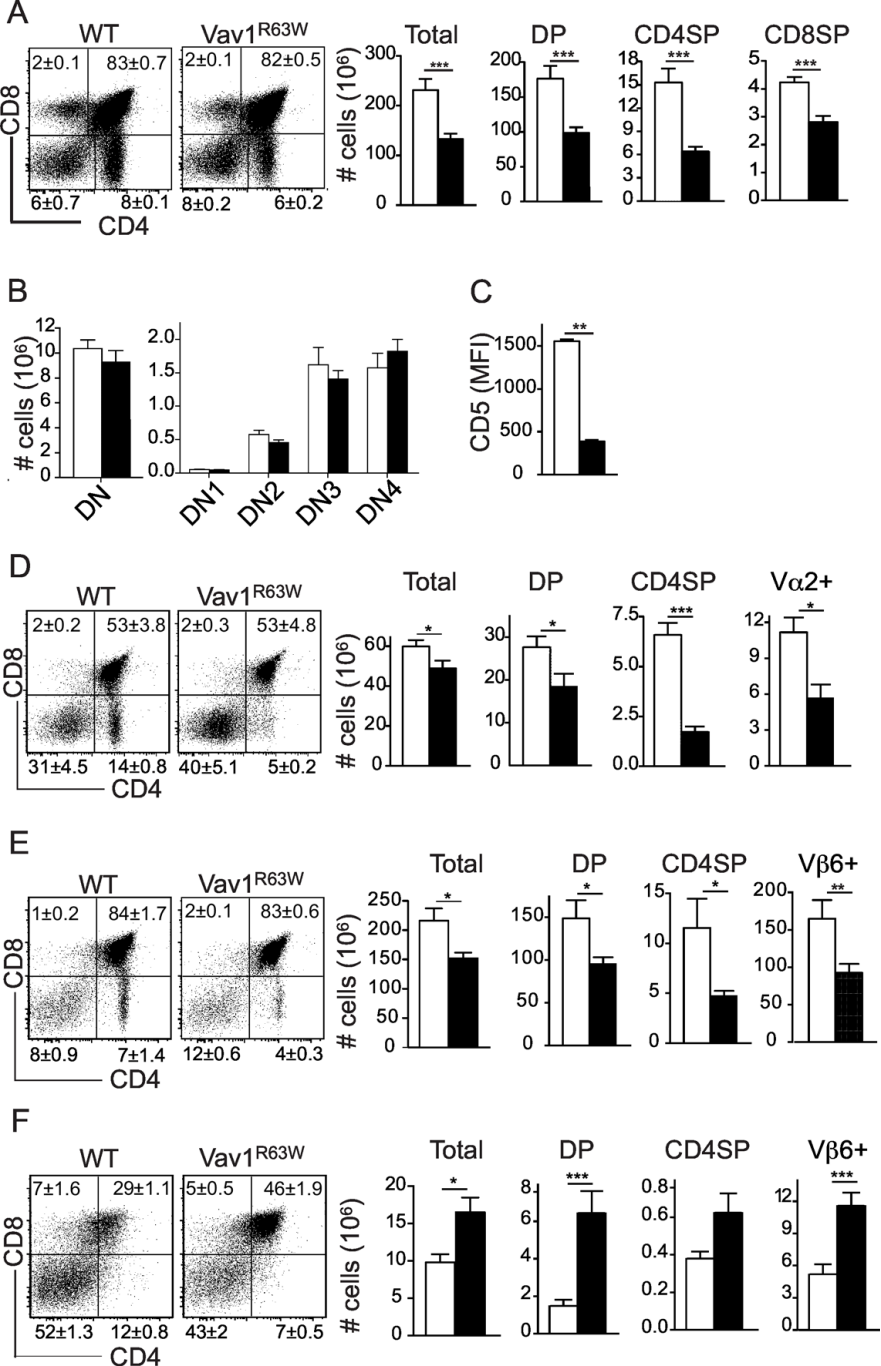
Impact of the Vav1^R63W^ mutation on T cell development. (A) Representative dot plots of CD4 and CD8 expression on thymocytes from WT (n = 16) and Vav1^R63W^ (n = 13) mice. Graphs represent the absolute numbers of total thymocytes and of each indicated population. (B) Mean absolute numbers of the different double negative (DN) populations in the thymus of WT (n = 5) and Vav1^R63W^ (n = 5) mice. (C) Mean fluorescence intensity (MFI) of CD5 expression on double positive (DP) thymocytes of WT (n = 16) and Vav1^R63W^ (n = 13) mice. (D) Representative dot plots of CD4 and CD8 expression on thymocytes of TCR transgenic OTII WT (n = 6) and OTII Vav1^R63W^ (n = 4) mice. Graphs show the absolute numbers of total thymocytes and of each indicated population. (E and F) Representative dot plots of CD4 and CD8 expression on thymocytes of female (E) and male (F) HY-TCR transgenic WT (n = 5 for females, n = 13 for males) and Vav1^R63W^ mice (n = 10 for females, n = 12 for males). Graphs show the absolute numbers of total thymocytes and of each indicated population. The values in dot plots represent the mean percentages of each population ± SEM. For A, C, E and F, the data represents the pool of three independent experiments. ■: Vav1^R63W^ mice; □: WT mice; *p≤0.05; **p≤0.01; ***p≤0.001.

The development of T cells in the thymus proceeds through a series of stages that are controlled by signals triggered by the pre-TCR and TCR complexes. We analyzed the impact of the Vav1^R63W^ polymorphism on these stages by using Vav1^R63W^ mice (S1 Fig). Vav1^R63W^ mice displayed a consistent lower cellularity in the thymus compared to that of wild-type littermates, with significantly fewer CD4^+^CD8^+^ double positive (DP), CD4^+^CD8^-^ (CD4SP) or CD4^-^CD8^+^ (CD8SP) single positive cells (Fig 1A). In contrast, Vav1^R63W^ mice had similar absolute numbers of CD4^-^CD8^-^ double negative (DN) thymocyte subsets (Fig 1B), suggesting normal pre-TCR signaling. The expression of CD5, a negative regulator that correlates positively with TCR signal intensity, was significantly lower on Vav1^R63W^ DP thymocytes (Fig 1C) indicating an impaired TCR signaling. To better investigate the positive selection of thymocytes towards the CD4 lineage, we crossed Vav1^R63W^ to OT-II transgenic mice, which express an ovalbumin-specific TCR restricted to MHC class II I-A^b^. We observed that the generation of thymocytes expressing the ovalbumin-specific TCR was impaired in Vav1^R63W^ mice (Fig 1D). Similar results were obtained using female HY-TCR transgenic mice whose Vβ6^+^ TCR recognizes a male-specific antigen presented by MHC class II I-A^b^ (Fig 1E). Next, we investigated the effect of the Vav1^R63W^ mutation on negative selection. As previously reported [16], male HY-TCR transgenic mice displayed a dramatic reduction of DP cells in the thymus due to early stage negative selection. In contrast, male HY-TCR transgenic mice in a Vav1^R63W^ background had twice as many thymocytes, resulting from increased DP cells (Fig 1F). These data indicate that negative selection is also impaired in Vav1^R63W^ KI mice. Collectively, our results reveal that Vav1^R63W^ has no major effect on the pre-TCR checkpoint but rather causes a defect in TCR-driven positive and negative selections of DP thymocytes.

### The Vav1^R63W^ polymorphism modifies T cell functions

**Fig 2:**
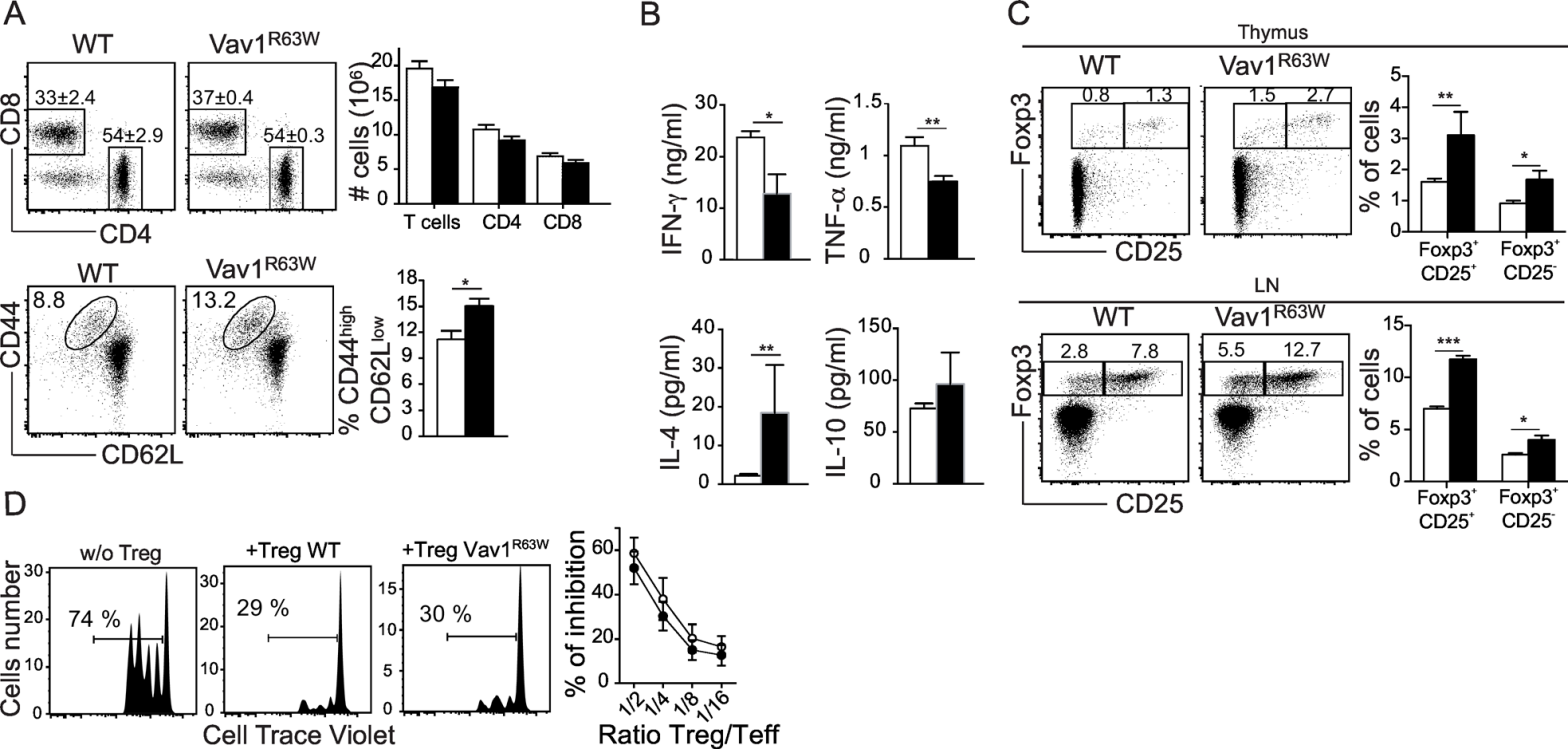
Impact of Vav1^R63W^ on T cell functions. (A, upper panels) Representative dot plots of CD4 and CD8 T cells in the lymph nodes (LN) of WT (n = 10) and Vav1^R63W^ (n = 8) mice. Graphs show absolute numbers of the indicated populations. (A, lower panels) Representative dot plots showing CD44 and CD62L expression on CD4 T cells in the LN of WT and Vav1^R63W^ mice. The histogram shows the mean percentages of activated CD4^+^CD44^high^CD62L^low^ cells. Data are representative of two independent experiments. (B) CD4^+^CD62L^high^ T cells from WT (n = 7) and Vav1^R63W^ (n = 7) mice were stimulated with anti-CD3 and anti-CD28 antibodies for 48h. Histograms show the mean concentration of IL-10, IL-4, IFN-γ and TNFα in the supernatants. (C) Representative analysis of the expression of Foxp3 and CD25 in CD4^+^ T cells in the thymus (upper panels) and the LN (lower panels) of WT (n = 10) and Vav1^R63W^ (n = 10) mice. Histograms show mean percentages of CD4^+^Foxp3^+^ CD25^+^ and CD4^+^Foxp3^+^CD25^-^ T cells. The data are from two independent experiments. (D) The suppressor activity of CD4^+^CD62L^+^CD25^bright^ Treg cells was measured using assays of suppression of T cell proliferation. Treg cells from WT or Vav1^R63W^ mice were co-cultured with WT effector T cells at various ratios. Results represent the % of inhibition and are representative of six independent experiments. ■: Vav1^R63W^ mice; □: WT mice; *p≤0.05; **p≤0.01; ***p≤0.001.

Although Vav1^R63W^ mice exhibit a defect in thymic development, there were no significant differences in the proportion and absolute numbers of CD4 and CD8 T cells in lymph nodes (Fig 2A) and spleen (S2A Fig). Yet, flow cytometry analysis of CD4 T cells from Vav1^R63W^ mice revealed a slightly higher frequency of CD44^high^CD62^low^ T cells, suggesting an increased in T cells with effector/memory phenotype. We hypothesized that this could be attributed to homeostatic proliferation—also known as lymphopenia-induced proliferation [17]—resulting from decreased thymic output. To determine whether the Vav1^R63W^ mutation affected effector CD4 T cell functions, we investigated the proliferation and cytokine production of sorted naïve CD4^+^CD62L^high^ T cells after stimulation with anti-CD3 and anti-CD28 mAbs *in vitro*. Proliferation analysis revealed only mild differences (S2B Fig). In contrast, CD4 T cells from Vav1^R63W^ mice produced less IFN-γ and TNFα, but more IL-4 (Fig 2B). We also examined the development of Treg cells in the lymphoid organs and observed a significant increase in the proportion of CD4^+^Foxp3^+^ Treg cells in the thymus, spleen and lymph nodes of Vav1^R63W^ mice (Figs 2C and S2C). We next investigated the impact of the Vav1^R63W^ mutation on the *in vitro* suppressive activity of CD4^+^Foxp3^+^ Treg cells. Freshly isolated CD4^+^CD62L^+^CD25^-^ naïve T cells were stained with cell trace violet and cultured with soluble anti-CD3 mAb and antigen-presenting cells for 3 days in the presence or absence of sorted CD4^+^CD62L^+^CD25^bright^ Treg cells. More than 95% of sorted CD4^+^CD62L^+^CD25^bright^ cells expressed Foxp3. Effector CD4 T cells stimulated in the absence of Treg cells proliferated readily and the immunosuppressive potential of Treg cells from Vav1^R63W^ mice was preserved at various Treg/Teff cell ratios (Fig 2D). Moreover, Treg cells from WT and Vav1^R63W^ mice expressed similar levels of Foxp3, CTLA-4, GITR and PD1 (S2D Fig). Thus, our results show that the Vav1^R63W^ mutation impacts on the cytokine production of effector T cells but not on the suppressive function of Treg cells *in vitro*.

### The Vav1^R63W^ reduces the susceptibility to CNS inflammation by impacting cytokine production by effector CD4 T cells

**Fig 3:**
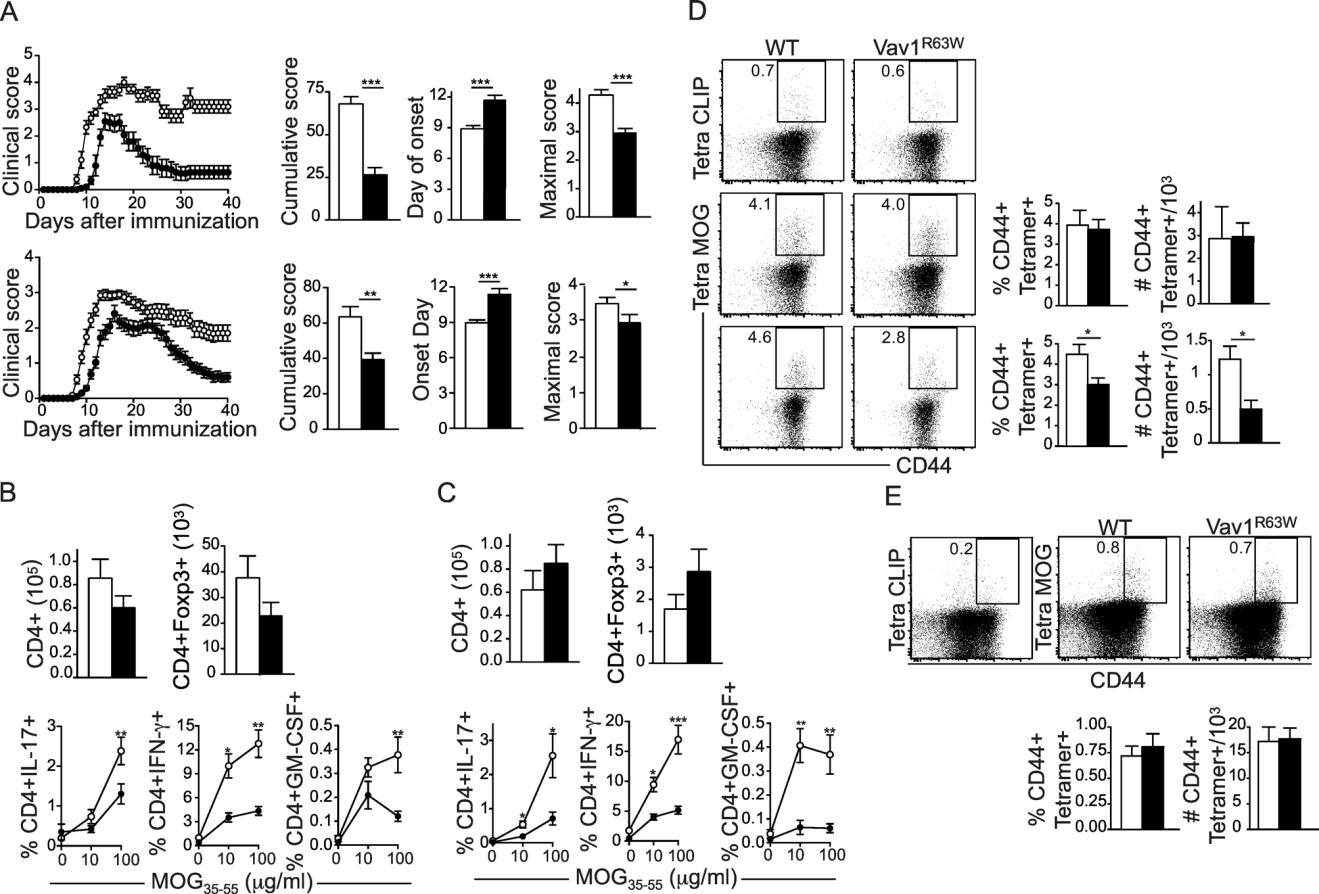
Vav1^R63W^ mice are less susceptible to EAE. (A) EAE was induced in mice by immunization with MOG_35-55_ peptide, using either 50 μg (upper panels, n = 10) or 100 μg (lower panels, n = 32). Disease severity was monitored daily. Histograms show mean cumulative scores, onset day and maximal score for the indicated genotypes. (B, C) Histograms show the mean absolute numbers of CD4 T cells and CD4^+^Foxp3^+^ Treg cells in the brain (B) and spinal cord (C) at the peak of the disease (day 15, n = 7 to 9 mice per group). Cells were re-stimulated overnight with MOG_35-55_ and cytokine expression was analyzed by intracellular staining in CD4 T cells from the brain (B) and spinal cord (C). (D, E) Tetramer staining showing CD4^+^CD44^+^ T cells specific for the MOG_35-55_ peptide in the brain (D, middle panels), spinal cord (D, lower panels) and LN (E) of WT (n = 6) and Vav1^R63W^ (n = 6) mice at day 15 after immunization. The tetramer staining showing CD4^+^CD44^+^ T cells specific for the CLIP peptide is used as control. Graphs show the mean percentages and absolute numbers of CD4^+^CD44^high^Tetramer^+^ cells for the indicated genotypes. Results are representative of two independent experiments. ■: Vav1^R63W^ mice; □: WT mice; *p≤0.05; **p≤0.01; ***p≤0.001.

**Fig 4:**
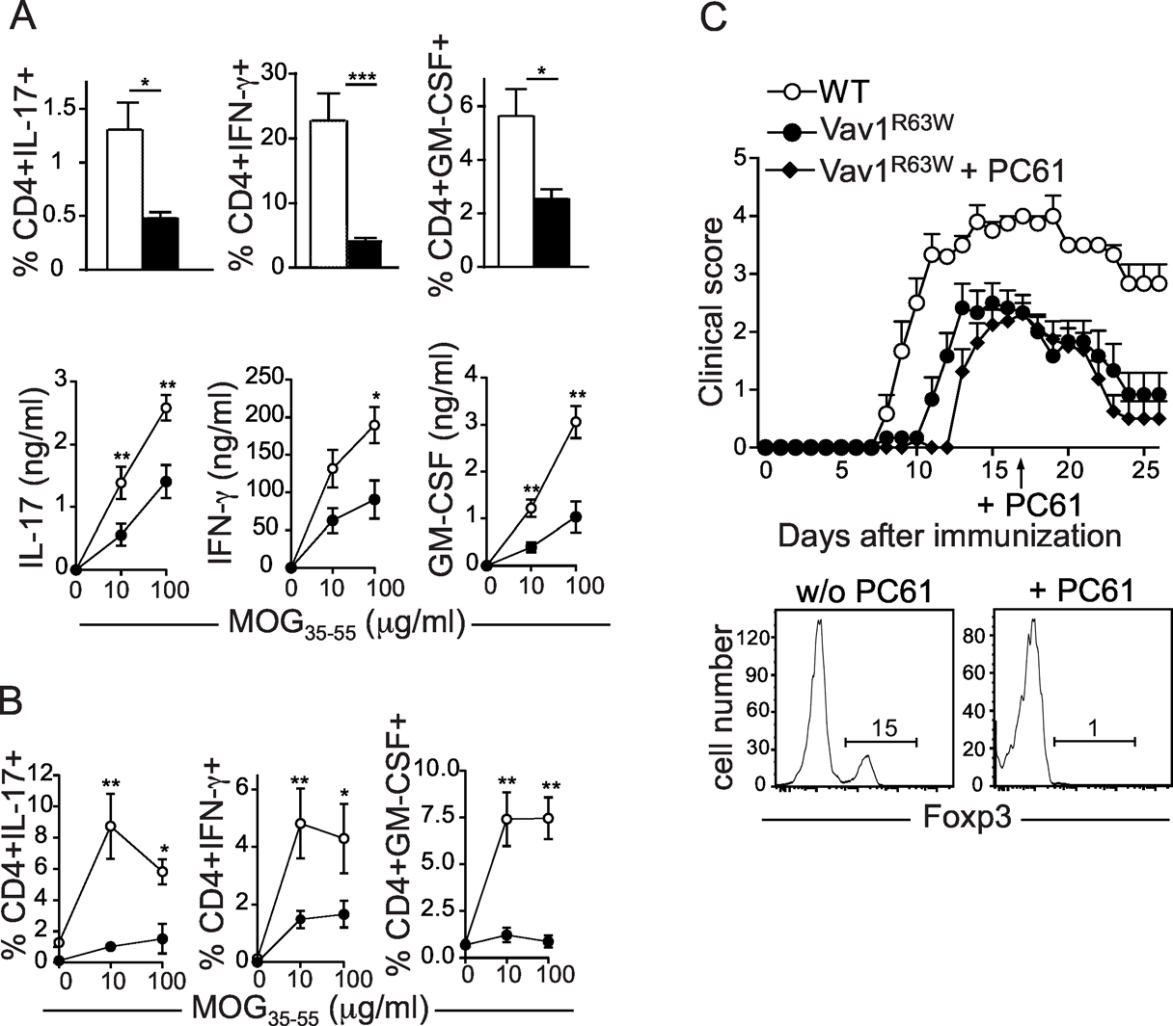
Reduced susceptibility to EAE in Vav1^R63W^ mice is due to an intrinsic defect in effector CD4 T cells. (A) LN cells from MOG_35-55_ immunized WT (n = 9) and Vav1^R63W^ (n = 8) mice were collected on day 15 after immunization and re-stimulated for 72 hours with MOG_35-55_ peptide. Graphs of the upper panels show intracellular cytokine expression by CD4^+^CD44^high^ cells after stimulation with 10 μg of MOG_35-55_. Lower panels show cytokine concentrations (IL-17, IFN-γ and GM-CSF) in the supernatants after stimulation with 10 or 100μg of MOG_35-55_ peptide. (B) Mixed bone marrow chimera were generated by transferring bone marrow from both CD45.1 WT and CD45.1 x CD45.2 Vav1^R63W^ into CD45.2 Vav1^R63W^ recipients (n = 6). 8 weeks later, the chimera were immunized with MOG_35-55_ peptide and sacrificed 15 days later for the analysis of cytokine expression by draining LN CD4 T cells. The graphs show the proportion of CD4 T cells originating from either the WT or Vav1^R63W^ bone marrow expressing IL-17, IFN-γ and GM-CSF after 72h of stimulation with MOG_35-55_. (C upper panel) EAE clinical scores analyzed after Treg cell depletion with PC61 i.p. injection at day 17 after MOG_35-55_ immunization (n = 10 for each group). (C lower panel) Representative analysis of the expression of Foxp3 in peripheral blood CD4^+^ T cells one week after PC61 administration. The data are representative of two independent experiments. ■: Vav1^R63W^ mice; □: WT mice; *p≤0.05; **p≤0.01; ***p≤0.001.

We previously reported that the Vav1^R63W^ polymorphism is fully included in the 1 cM locus controlling susceptibility to CNS inflammation in rats [15]. To formally demonstrate the implication of the Vav1^R63W^ polymorphism in this phenotype, WT and Vav1^R63W^ mice were immunized with 50 or 100 μg of MOG_35-55_ peptide. Although the incidence of the disease was similar between the two groups, Vav1^R63W^ mice developed a less severe disease, with delayed onset and quicker recovery resulting in reduced clinical scores (Fig 3A). The analysis of CNS infiltration at day 15 and 30 after MOG_35-55_ immunization revealed no significant differences in the numbers of CD4 T cells or CD4^+^Foxp3^+^ T cells infiltrating the brain (Figs 3B and S3A) or the spinal cord (Figs 3C and S3B), thereby excluding a defect in CD4 T cell migration or increased Treg numbers as the basis of reduced disease severity. However, the CNS-infiltrating CD4 T cells from both the brain (Fig 3B) and spinal cord (Fig 3C) of MOG_35-55_-immunized Vav1^R63W^ mice produced significantly less IFN-γ, IL-17 and GM-CSF. Similar results were obtained in draining lymph nodes (Fig 4A). Using tetramer staining, we showed similar frequencies of MOG_35-55_-specific CD4 T cells in the brain (Fig 3D) and draining LNs (Fig 3E) of WT and Vav1^R63W^ mice, although a moderate decrease was observed in the spinal cord of Vav1^R63W^ mice (Fig 3D). This suggests that the reduced cytokine expression is not the consequence of impaired development or expansion of MOG-specific CD4 T cells. We next examined whether the defect in cytokine production observed in Vav1^R63W^ mice is intrinsic to effector CD4 T cells or is the consequence of either increased Treg frequency or modified function of other immune cells such as APCs. For this purpose, we generated mixed bone marrow chimeras by transferring a 1:1 mixture of bone marrow from WT mice bearing the congenic CD45.1 marker and Vav1^R63W^ mice bearing both CD45.1 and CD45.2 congenic markers into irradiated lymphopenic CD45.2 recipient mice. These chimeras revealed that the cytokine profile of Vav1^R63W^ CD4 T cells upon MOG_35-55_ immunization was not influenced by the presence of hematopoietic cells from WT mice (Fig 4B). In addition, we showed that reduced EAE course in Vav1^R63W^ mice was not changed by the depletion of Treg by one single injection at day 17 of the anti-CD25 PC61 mAb which depletes Tregs (Fig 4C). Altogether, our results highlight the key role of Vav1 in the pathophysiology of EAE and suggest that the Vav1^R63W^ polymorphism protects against the development of CNS inflammation by reducing the production of encephalitogenic cytokines by autoreactive CD4 T cells.

### The Vav1^R63W^ mutation reduces Vav1 adaptor functions

**Fig 5:**
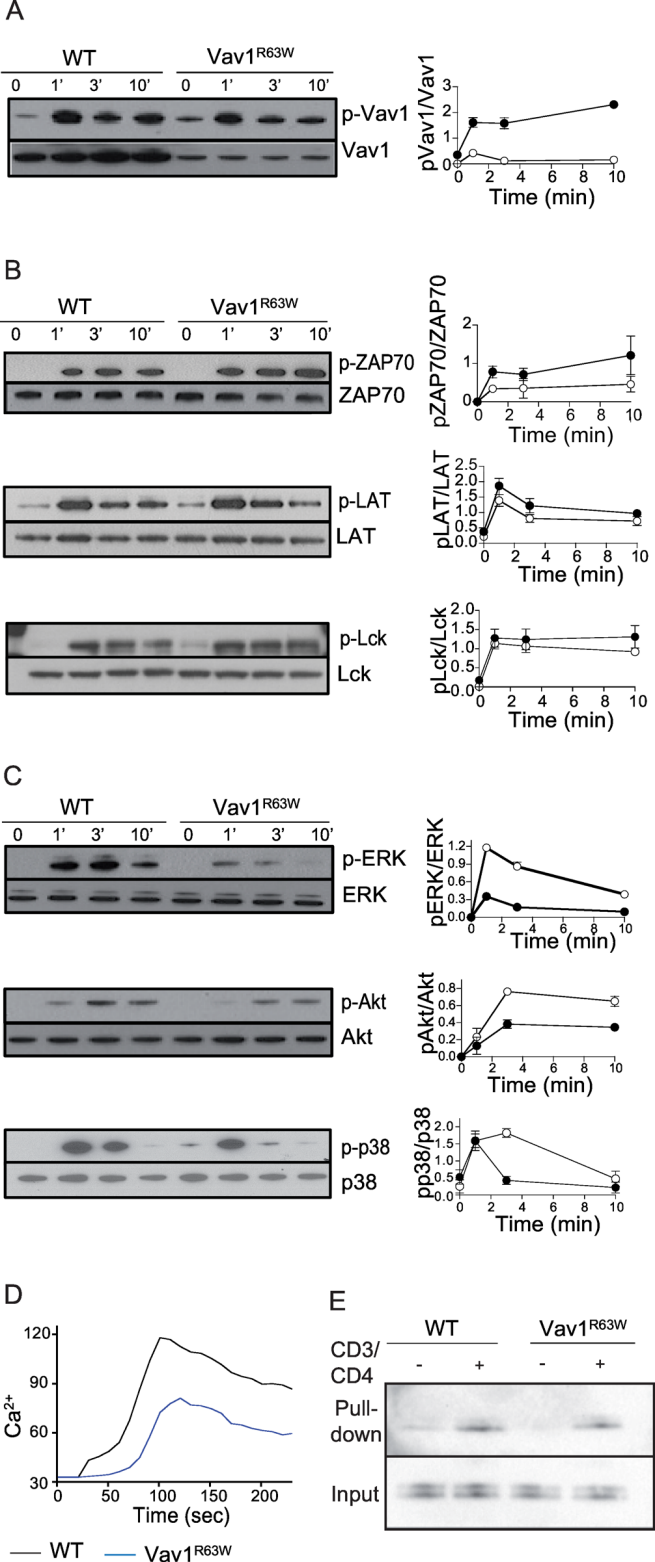
Impact of the Vav1^R63W^ mutation on Vav1 expression, functions and TCR signaling. Phosphorylation of Vav1 (A), ZAP-70, LAT and LcK (B), Erk, Akt and p38 (C) were analyzed by western blot in CD4 T cells after anti-CD3 and anti-CD4 stimulation for the indicated times. The graphs show the relative abundance of p-Vav1, p-ZAP-70, p-LAT, pLcK, p-Akt, p-Erk and p-p38. (D) Analysis of Ca^2+^ influx in purified CD4 T cells from WT and Vav1^R63W^ mice loaded with Indo-1 following stimulation with anti-CD3 mAb. Data are representative of three independent experiments. (E) Analysis of Rac1 activation in WT and Vav1^R63W^ CD4 T cells stimulated or not with anti-CD3 and anti-CD4 mAbs using pull-down assay to detect the relative amounts of Rac1-GTP. Total Rac1 levels were used as loading controls. Data are representative of three independent experiments. ■: Vav1^R63W^ mice; □: WT mice.

We next investigated which Vav1-dependent TCR signaling pathways were affected by the Vav1^R63W^ mutation. We first showed that the Vav1^R63W^ variant was highly phosphorylated, together with a 75% reduction of its expression at the protein level (Fig 5A). Vav1^R63W^ had, however, no impact on proximal TCR signaling, as revealed by normal phosphorylation levels of ZAP70, LAT and Lck (Fig 5B). In contrast, this variant strongly impaired Vav1-dependent distal TCR signaling, as evidenced by a significant reduction in the phosphorylation of Erk, Akt and p38 (Fig 5C). Further, this was associated with reduced calcium flux after TCR engagement (Fig 5D). In contrast, the TCR induced activation of Rac1 was normal in Vav1^R63W^ mice, suggesting normal GEF activity (Fig 5E). Therefore, the biological effects of the Vav1^R63W^ variant are likely to be mediated by the reduction in Vav1 protein expression and the consequent decrease in Vav1 adaptor functions.

## Discussion

In the present study, we analyzed the impact of the recently identified Vav1^R63W^ variant on the development and functions of T cells, as well as on the susceptibility to CNS neuroinflammation. In contrast to Vav1 deficiency [1, 3, 5], we found that the Vav1^R63W^ variant had only mild effects on thymic development of T cells and on T cell homeostasis in the periphery. In addition, the Vav1^R63W^ KI mice were less susceptible to CNS inflammation, resulting from a reduced production of inflammatory cytokines (IFN-γ, IL-17 and GM-CSF) by autoreactive CD4 T cells. Despite an increased proportion of Foxp3 Treg cells in Vav1^R63W^ mice, the reduction in cytokine production was intrinsic to effector CD4 T cells and depletion of Treg cells had no impact on EAE development. Finally, we showed that Vav1^R63W^ had normal GEF activity but reduced adaptor functions. Together, the analysis of this natural Vav1 variant formally established for the first time that Vav1 adaptor functions are essential for both T cell functions and susceptibility to autoimmune neuroinflammation.

The interaction between the TCR and MHC-peptide complexes leads to the initiation of TCR signaling and represents a key step for the orchestration of the adaptive immune response. Indeed, the intracellular signaling pathways triggered upon TCR engagement finely control the thymic ontogeny of T cells, the mature T cell differentiation, expansion and activation [18, 19, 20]. In the thymus, engagement of the pre-TCR leads to the differentiation of the most immature DN thymocytes towards the DP stage, through a selection process called β-selection. Next, DP thymocytes expressing a mature TCRαβ and displaying low avidity for self-peptide-MHC complexes undergo a process of positive selection into either MHC class II-restricted CD4^+^CD8^-^ or MHC class I-restricted CD4^-^CD8^+^ SP cells. Conversely, DP thymocytes that have a TCR with high avidity for self-peptide-MHC are eliminated by negative selection. The outcome of these different selection events is critically dependent on TCR signaling. Studies of Vav1-deficient mice have shown that the development of T cells is partially blocked at the pre-TCR checkpoint in the thymus, leading to a strongly block of both positive and negative selections [1, 3, 5]. In contrast, our results reveal that Vav1^R63W^ has no major effect on the pre-TCR checkpoint, but rather causes a defect in TCR-driven positive and negative selections of DP thymocytes. In agreement, the critical downstream mediators of Vav1 signaling in response to TCR stimulation such as Ca^2+^ flux and activation of Erk, p38 and Akt were reduced in Vav1^R63W^ CD4 T cells. In contrast, the phosphorylation of upstream signaling molecules such as LAT, LCK and ZAP70 was not affected. Since knock-in mice carrying a GEF-deficient Vav1 mutant revealed that the GEF activity of Vav1 is dispensable for Ca^2+^ flux and Erk activation [11], our results suggest that Vav1^R63W^ exhibits a defect in its adaptor functions. In contrast, the TCR induced activation of Rac1 was normal in Vav1^R63W^ mice. Thus, this study highlights the essential roles of Vav1 adaptor functions in TCR induced positive and negative selections and its minor role in pre-TCR β-selection.

We previously reported that the difference in susceptibility to EAE in rats was genetically controlled by a locus of 1 cM in chromosome 9 that contains the Vav1^R63W^ polymorphism [14, 15 ]. The present study using Vav1^R63W^ KI mice provides the definitive demonstration that this mutation *per se* leads to a reduced severity of EAE. The analysis of CNS infiltration revealed no major differences in numbers of infiltrating CD4 T cells, thereby excluding the hypothesis that the reduced neuroinflammation observed in Vav1^R63W^ KI mice may be the result of a defect in autoreactive CD4 T cell numbers or migration. Rather, the CD4 T cells from MOG_35-55_ immunized Vav1^R63W^ KI mice produced significantly less inflammatory cytokines such as IFN-γ, IL-17 and GM-CSF. This defect likely originates in the periphery since similar results were obtained in draining lymph nodes. The analysis of MOG_35-55_-specific CD4 T cells using tetramer staining suggests that the reduced cytokine expression is not the consequence of impaired development or expansion of MOG-specific CD4 T cells. By using mixed bone marrow chimeras, we showed that the defect of cytokine production was intrinsic to effector Vav1^R63W^ KI CD4 T cells and was not the consequence of either increased Treg cell frequency or modified function of other immune cells such as APCs. Consequently, depletion of Treg in Vav1^R63W^ KI mice has no impact on EAE severity. This defect in cytokine expression is particularly relevant when considering that several studies have established that IFN-γ, IL-17 and GM-CSF are the main effector cytokines in the pathophysiology of both EAE and multiple sclerosis [21–23]. In addition, our findings are fully in line with our previous study using cohorts of patients with multiple sclerosis, in which we demonstrated a strong association between Vav1 expression, susceptibility to multiple sclerosis and production of inflammatory cytokines by CD4 T cells [15]. Our results, however, contrast with data using Vav1-deficient CD4 T cells or CD4 T cells harboring a mutated Vav1 with defective GEF activity, which rather showed increased production of IFN-γ [11, 24 ]. The signaling pathway that depends on Vav1 adaptor function, therefore, plays an important role in Th1/Th17 differentiation and could be targeted for immunomodulation of immune mediated diseases.

The differentiation of naïve CD4 T cells into functionally polarized T helper cell subsets depends notably on the strength of TCR-dependent signaling pathways upon antigen recognition [19]. In general, weak TCR signaling leads to transient Erk activation and favors Th2/Treg differentiation, whereas stronger TCR signaling leads to sustained Erk activation and favors Th1/Th17 differentiation [19, 25, 26]. Our findings are in line with these data, since we showed that Vav1^R63W^ leads to a reduction of TCR signaling as revealed by a decrease of calcium flux and of Erk, Akt and p38 activities, which was associated with a decreased production of IFN-γ, IL-17 and GM-CSF by CD4 T cells. Of note, it was shown that Erk inhibitors could attenuate EAE by suppressing autoantigen-specific Th17 and Th1 responses [27]. Moreover, the genetic ablation of Erk2 impedes Th1 differentiation, while enhancing the development of induced Treg [28]. Concerning the p38 MAPK pathway, it has been shown that a single copy disruption of the *p38* gene or a p38 inhibitor markedly reduce the pathogenesis of EAE by decreasing IL-17 production [29, 30]. In contrast, the role of Akt-dependent pathways and calcium flux in CD4 T cell differentiation remains contradictory [31–35]

The capacity to produce knockout mice has dramatically accelerated our knowledge on the immunological consequences of the complete loss of critical components of the TCR signaling pathways. However, in humans, the largest source of genetic variation is rather composed of single-nucleotide substitutions, for which it is far more difficult to predict their physiological consequences. Importantly, these can often affect biological pathways in unpredictable ways, as revealed recently by the use of mouse models with hypomorphic variants for SLP76, LAT and Zap70 [36–39]. Our study reveals that the Vav1^R63W^ model described herein is instrumental to expand our understanding of the immunological consequences of genetic variations of Vav1 expression and function. This model highlights the importance of Vav1 adaptor functions in the differentiation of CD4 T cells into Th1/Th17 subsets and suggest that genetic or acquired alterations in Vav1 signaling could play a major role in susceptibility to the many immune-mediated diseases, including autoimmune diseases where Th1/Th17 play a preponderant role.

## Materials and Methods

### Generation of Vav1^R63W^ knock-in mice

Vav1 protein is evolutionarily conserved from nematodes to mammals and the analysis of rat and mouse Vav1 sequences revealed 98% homology. Comparative genomics studies indicated that the arginine found at position 63 in LEW rats is highly conserved among species while the tryptophan at this position is peculiar to BN rats. A genomic fragment containing exon 1 of the *Vav1* gene was isolated from a BAC clone of C57BL/6 origin. The CGG codon found in exon 1 of the *Vav1* gene and coding for the arginine residue present at position 63 of Vav1 was converted into a TGG codon coding for a tryptophane. A *lox*P-tACE-CRE-PGK-gb2-*neo*r-*lox*P cassette (NEO; [38]) was introduced in the intron separating exons 1 and 2 of the *Vav1* gene (S1A Fig). After electroporation of JM8.F6 C57BL/6N ES cells [40] and selection with G418, colonies were screened for homologous recombination by Southern blot. The 3’ single-copy probe corresponded to a 582 bp genomic fragment located in intron 1 (denoted 3’ probe in S1A Fig). When tested on *BglI* digested DNA, it hybridizes to a 16.4 kb wild-type fragment or to a 10.2 kb recombinant fragment. The occurrence of an appropriate homologous recombination event at the 5’ side was screened by PCR using the following oligonucleotides: 5’-AAACCTAGTGGGCGCTCTCCA-3’ and 5’-TGACGAGTTCTTCTGAGCGG-3’. This pair of primer amplifies a 3791 bp fragment. Finally, a neomycin-specific probe was used to ensure that adventitious non-homologous recombination events had not occurred in the selected ES clones. Mutant ES cells were injected into FVB blastocysts. Germline transmission led to the self-excision of the NEO selection cassette in male germinal cells. Screening of mice for the presence of the *Vav1*^R63W^ mutation was performed by PCR using the following oligonucleotides 5’-TGTAGGGGGCATCTGTCTGTCTG-3’ and 5’-AAATACCCTGGAGACTGCAGCAG-3’. This pair of primers amplifies a 203 bp band in the case of the wild-type allele and a 269 bp band in the case of the *Vav1*^R63W^ allele (S1C Fig). Mice homozygous for Vav1^R63W^ were fertile, indicating that the Vav1^R63W^ mutation did not affect the embryonic development or viability of the KI mice. Mice harboring the *Vav1*^R63W^ mutation (international strain designation C57BL/6-*Vav1*^tm2Mal^) were kept on a C57BL/6 background. OVA-specific OT-II-TCR transgenic mice [41] and HY-TCR transgenic mice, whose CD4 T cells are specific for HY peptide presented by IA^b^ [16] were kindly provided by Dr. Sylvie Guerder (Centre de Physiopathologie Toulouse-Purpan) and were backcrossed with Vav1^R63W^ mice. All mice were housed under specific pathogen-free conditions at the INSERM animal facility (Zootechnie UMS-006; accreditation number A-31 55508), which is accredited by the French Ministry of Agriculture to perform experiments on live mice. All experimental protocols were approved by the local ethics committee and are in compliance with the French and European regulations on care and protection of the Laboratory Animals (EC Directive 2010/63).

### Antibodies for flow cytometry and Elisa

The mAbs used for flow cytometry were as follows: RM4-5 (anti-mouse CD4), 53–6.7 (anti-mouse CD8α), IM7 (anti-mouse CD44), PC61 (anti-mouse CD25), MEL-14 (anti-mouse CD62L), H57-597 (anti-mouse TCR αβ), FJK-165 (anti-mouse Foxp3), 53–7.3 (anti-mouse CD5), A20 (anti-mouse CD45.1), 104 (anti mouse CD45.2), anti-mouse IL-17A, anti-mouse GM-CSF, anti-mouse IFN-γ. The fluorescent conjugated antibodies were purchased from e-Biosciences, BD Biosciences and Biolegend. Antibodies used for ELISA were: 11B11 (anti-IL-4), AN18 (anti-IFN-γ), purified anti-mouse IL-17A, purified anti-mouse GM-CSF, Biotin anti-mouse IFN-γ (XMG1.2), Biotin anti-mouse IL-17A, Biotin anti-mouse GM-CSF. These antibodies were purchased from BD Biosciences. The BVD6-24G2 (anti-mouse IL-4 Biotin) is from e-Biosciences.

### Induction of experimental autoimmune encephalomyelitis (EAE)

The MOG_35–55_ (MEVGWYRSPFSRVVHLYRNGK) peptide was purchased from Polypeptide Laboratories (San Diego, CA) with a purity grade >95%. 8–12 week-old mice were immunized subcutaneously at the base of the tail with 50 μg or 100 μg of MOG_35-55_ peptide emulsified in CFA (BD Difco, Franklin Lakes NJ) containing 500 μg of *Mycobacterium tuberculosis* (Strain H37, Difco). For active EAE, mice were injected intravenously with 200 ng of pertussis toxin (List Biological Laboratories, Campbell, CA) at days 0 and 2 post-immunization. Clinical scores were recorded daily as follow: 0, no signs of disease; 1, loss of tone in the tail; 2, hind limb paresis; 3, hind limb paralysis; 4, tetraplegia; 5, moribund. Intraperitoneal injection with anti-CD25 mAb (PC61) (500 μg/ml) antibody was performed at day 17 after MOG_35-55_ peptide immunization.

### Isolation and functional characterization of CNS mononuclear cells

Mice were anesthetized with Ketamine and perfused with cold PBS. Brain and spinal cord were collected separately, homogenized and digested with collagenase D (2.5 mg/ml, Roche Diagnostics), Dnase I (10 μg/ml) and TLCK (1 μg/ml, Roche, Basel, Switzerland) for 30 min at 37°C. Cells were then washed, suspended in 37% Percoll, and layered on 70% Percoll. After a 20-minute centrifugation at 2000 rpm, the mononuclear cells were collected from the interface, washed and resuspended in culture medium. Isolated cells were counted using a hematometer and then stained in order to analyze the presence of different cell populations by flow cytometry. 3x10^5^ CNS infiltrated cells were stimulated O/N with different concentrations of MOG_35-55_ (0, 10 and 100 μg) to analyze the cytokine expression by CD4 T cells using intracellular staining (ICs). Similarly LN cells and splenocytes were stimulated with different concentrations of MOG_35-55_ (0,10 and 100 μg) for 72 hours to investigate the cytokine expression using intracytoplasmic staining and ELISA. MOG_38-49_-I-A^b^ and CLIP-I-A^b^ tetramers were obtained from the NIH tetramer core facility (Emory University, Atlanta, USA). Cells from the brain, spinal cord and LNs were incubated with tetramers for 2h at room temperature at a concentration of 0.03 mg/ml and then stained for surface markers for flow cytometry analysis.

### Mixed bone marrow chimera and transfer experiments

CD45.2 Vav1^R63W^ recipient mice were irradiated (9.5 Gy) the day before i.v. injection of a 1:1 mixture of bone marrow cells. Cells were harvested after flushing cells from tibias and femurs and 20x10^6^ cells from WT (CD45.1) and Vav1^R63W^ (CD45.1xCD45.2) were injected per mouse. The control groups received BM from either WT or Vav1^R63W^ mice. 8 weeks later, the chimeras were immunized with MOG_35-55_ and the cytokine profile of MOG-specific donor cells was analyzed by intracytoplasmic staining on day 15 after immunization.

### Purification of CD4 T cell subsets and Treg cell suppression assay

To purify naïve CD4^+^CD62L^+^ T cells, CD4 T cells were negatively selected using Dynal cocktail antibodies supplemented with PC61 (an anti-CD25 mAb) to eliminate Treg cells. Cells were then positively selected with anti-CD62L beads on MS columns (Miltenyi, Auburn CA) according to the manufacturer’s instructions. For functional test, naïve CD4 T cells were stimulated with plate-bound anti-CD3 (3 μg/ml) and soluble anti-CD28 (1 μg/ml). Cytokine production was analyzed by ELISA 48h hours after culture. For Treg suppression assays, CD4 T cells were stained with antibodies directed against TCR, CD4, CD62L and CD25. CD4^+^CD62L^+^CD25^-^ (effector T cells) and CD4^+^CD62L^+^CD25^bright^ (regulatory T cells) were sorted using FACSAria SORP (BD Biosciences). The purity of these populations was higher than 95%. Effector and regulatory T cells were co-cultured at different ratio (Treg:Teff, 1:1, 1:2, 1:4, 1:8, 1:16) in the presence of WT irradiated splenic antigen presenting cells and soluble anti-CD3 (1 μg/ml). Effector cells were stained with cell trace violet (life technologies) in order to track their proliferation. Proliferation was analyzed 3 days after culture by flow cytometry.

### Cytokine measurement

Enzyme immunoassays were used to measure cytokines in culture supernatants. 96 well plates were coated for 2 h at 37°C with anti-IFN-γ, anti-IL-17 or anti-GM-CSF in carbonate buffer 0.05 M pH 9.6. Culture supernatants or standards were incubated 2 h at 37°C. The plates were then incubated for 1h30 min with a secondary biotinylated antibody specific for each cytokine, followed by 20 min incubation with streptavidin-phosphatase alkaline at 37°C. Finally, plates were revealed by phosphatase alkaline substrate and absorbance was measured at 450/540 nm. For intracellular cytokine staining, cells were stimulated with different concentrations of MOG_35-55_ (0, 10 and 100 μg) and treated with monensin (Golgiplug 1 μg/ml, BD Biosciences) at 37°C, in a humidified 5% CO2 atmosphere for 4 h. After staining of surface markers (TCR, CD4 and CD44), cells were fixed and permeabilized with Cytofix/Cytoperm and Perm/Wash buffer (e-Biosciences) according to the manufacturer’s instructions. Cells were then incubated with antibodies recognizing cytokines (IL-17, IFN-γ, GM-CSF) or isotype controls for 20 min and washed twice with Perm/Wash buffer before analysis. The production of IL-4, TNFα, IL-10 and IFN-γ by naive CD4 T cells was assayed using Cytometric Bead Array cytokine kit (BD Biosciences).

### Analysis of TCR signaling in CD4 T cells

Purified CD4 T cells were stimulated at 37°C in non-supplemented RPMI 1640 using preformed complexes of biotinylated anti-CD3 (clone 145-2C11, Biolegend, 30 μg/ml /10^7^ cells), anti-CD4 (GK1.5, Biolegend, 30 μg/10^7^ cells) and streptavidin (15 μg/10^7^ cells). Stimulation was stopped by the addition of twice-concentrated lysis buffer (100 mM Tris, pH 7.5, 270 mM NaCl, 1 mM EDTA, 20% glycerol and 0.2% n-dodecyl-β-maltoside) supplemented with protease and phosphatase inhibitors. After 10 min of incubation on ice, cell lysates were centrifuged at 20,000 x *g* for 15 min at 4°C. The protein concentration was first assessed using bradford assay and normalized. Proteins were then denatured in Laemmli buffer and analyzed by SDS-PAGE followed by Western blotting on PVDF membranes (Immobilon). ECL Prime (Amersham) was used as revelation substrate. Signal intensity quantification was performed using ImageJ software (1.47v). Two loadings were performed simultaneously for each time point, one for phosphoproteins and the other for total proteins. A GAPDH staining was also performed on each blot as loading control. The blots were then scanned, bands of interest were quantified and background line was subtracted. To determine specific phosphorylation, the signal from phosphorylated bands was divided by the appropriate loading control and all values were expressed as a fold increased of bands intensity. Signals below detection were set at 0. The antibodies used for biochemical studies were, anti-Vav1 (C-14) and anti-P-Vav1 Tyr174 From Santa Cruz Biotechnology, anti-LAT (1D-1) from Thermo Scientific, anti-P-LAT (pY226) from BD Pharmingen, anti-Akt, anti-P-Akt Ser473 (D9E), anti-P-Erk1/2 Thr202/Tyr204 (D13.14.4E), anti-Erk (3A7), anti ZAP-70 (D1C10E), anti-P-ZAP70 (Tyr319), HRP-linked anti-rabbit IgG, HRP-linked anti-mouse IgG, and HRP-linked anti-rabbit IgG from Cell Signaling Technologies.

Rac1 pull-down assays were performed using Rac1 activation Assay Biochem Kit (Cytoskeleton) following the manufacturer’s instructions.

### Calcium flux analysis

For Calcium flux analysis, CD4 T cells were loaded at 37°C for 30 min with the fluorescent calcium indicator Indo-1 (Invitrogen) at 5 μM. The cells were then washed and stained with surface markers (TCR and CD4). Ca influx was measured by flow cytometry using an LSRII (BD Bioscience). Cells were incubated with 0.5 μg/ml of biotylinated anti-CD3 mAb (145-2C11) at 37°C for 30 sec. Medium were then added and baseline level was measured for 30s, at which time streptavidin (Sigma-Aldrich) was added at 1 mg/ml as a cross-linker. Finally ionomycin was added at 2 μg/ml to all samples to verify Indo-1 labeling. The relative concentration of Ca was measured as the ratio between the Ca-bound dye and the Ca free dye. Data were analyzed using FlowJo software.

### Data analysis

Data are expressed as mean ± s.e.m. The GraphPad Instat statistical package was used for statistical analyses (GraphPad Software, Inc., La Jolla, CA USA). Results were compared using Mann–Whitney test. Results were considered statistically significant when the p value was <0.05. *: p<0.05; **: p<0.01; ***: p<0.001.

## Supporting Information

S1 Fig Generation and validation of Vav1^R63W^ knock-in mice. (A) Schematic drawing of the wild-type Vav1 allele before targeting, the targeting vector, the Vav1^R63W^neo targeted allele generated in embryonic stem (ES) cells and the Vav1^R63W^ allele with a single LoxP site after deletion of the Cre/Neo selection cassette following germline transmission. The targeting vector contains sequences encompassing exon 1 of the *Vav1* gene. A mutation giving rise to the intended R63W substitution was introduced in exon 1 and a Cre-Neo selection cassette flanked by two LoxP sites was introduced in intron 1. Introduction of the neo gene into intron 1 was identified by Southern blotting of genomic DNA cut by BglI and probed with a 3’probe in intron 1. Predicted size of hybridizing bands are shown. The occurrence of an appropriate homologous recombination event at the 5’ side was screened by PCR using the following oligonucleotides: 5’-AAACCTAGTGGGCGCTCTCCA-3’ and 5’-TGACGAGTTCTTCTGAGCGG-3’. Black boxes represent exon 1 and 2 of Vav1, grey triangles represent LoxP sites, green box shows the location of the 3’ single-copy probe and blue box that of the PCR amplicon allowing to probe for proper recombination events at the 5’ end. ES clones containing the R63W allele were injected into FVB blastocysts to generate chimeric mice. Successful germline transmission was confirmed by sequencing (B) and PCR (C) with 5’-TGTAGGGGGCATCTGTCTGTCTG-3’ and 5’-AAATACCCTGGAGACTGCAGCAG-3’. This pair of primers amplifies a 203 bp band in the case of the wild-type allele and a 269 bp band in the case of the Vav1^R63W^ allele.
(TIF)

S2 Fig Impact of Vav1^R63W^ on T cell phenotype and functions. (A, upper panels) Representative dot plots of CD4 and CD8 T cells in the spleen of WT (n = 10) and Vav1^R63W^ (n = 8) mice. The values on each cytometry profile represent the mean percentages of each population (mean ± SEM). Graphs show absolute numbers of each indicated population. (A, lower panels) Representative flow cytometry dot plots showing CD44 and CD62L expression on CD4 T cells in the spleen of WT and Vav1^R63W^ mice. Graphs show the mean percentages of activated CD4^+^CD62L^low^CD44^high^ population. (B) Naïve CD4^+^CD62L^high^ T cells were purified from WT (n = 5) and Vav1^R63W^ (n = 5) mice, stained with cell trace violet and stimulated with anti-CD3 and anti-CD28 antibodies for 72h. Proliferation of CD4 T cells was then analyzed by flow cytometry. Histograms represent the percentage of proliferating CD4 T cells. Graphs represent the percentage of non divided cells, cells divided one or two times and cells divided more than 3 times for the indicated genotypes. (C) Representative flow cytometry profiles of Foxp3+ T cells gated on CD4^+^ T cells in the spleen of WT (n = 10) and Vav1^R63W^ (n = 8) mice. Graphs show mean percentages of CD4^+^Foxp3^+^CD25^+^ and CD4^+^Foxp3^+^CD25^-^ T cells in the spleen. (D) Graphs represent the expression of characteristic markers by CD4^+^Foxp3^+^ T cells in the spleen of WT (n = 5) and Vav1^R63W^ (n = 5) mice. ■: Vav1^R63W^ mice; □: WT mice; *p≤0.05; ***p≤0.001.
(TIF)

S3 Fig Reduced severity to EAE in Vav1^R63W^ mice is associated with a defect in effector CD4 T cells. At day 30 after immunization, mononuclear cells were isolated from the CNS of individual mice (n = 7 per group). Graphs show the mean absolute numbers of CD4 T cells and CD4 Foxp3 Treg cells in the brain (A) and spinal cord (B). (C) Total LN cells collected on day 30 after immunization were re-stimulated for 72 hours with MOG_35-55_ peptide, graphs of the upper panels show cytokine expression by CD4^+^CD44^high^ cells using intracellular staining after stimulation with 10 μg of MOG_35-55_. Lower panel show cytokine concentrations (IL-17, IFN-γ and GM-CSF) in the supernatants after stimulation with MOG_35-55_ peptide (10 or 100 μg). ■: Vav1^R63W^ mice; □: WT mice; **p≤0.01
(TIF)
