# Supplementary figures and images for: A Natural Variant of the T Cell Receptor-Signaling Molecule Vav1 Reduces Both Effector T Cell Functions and Susceptibility to Neuroinflammation
Source: PLoS Genet. 2016 Jul 20;12(7):e1006185. doi: 10.1371/journal.pgen.1006185 (PMC4954684; doi:10.1371/journal.pgen.1006185)

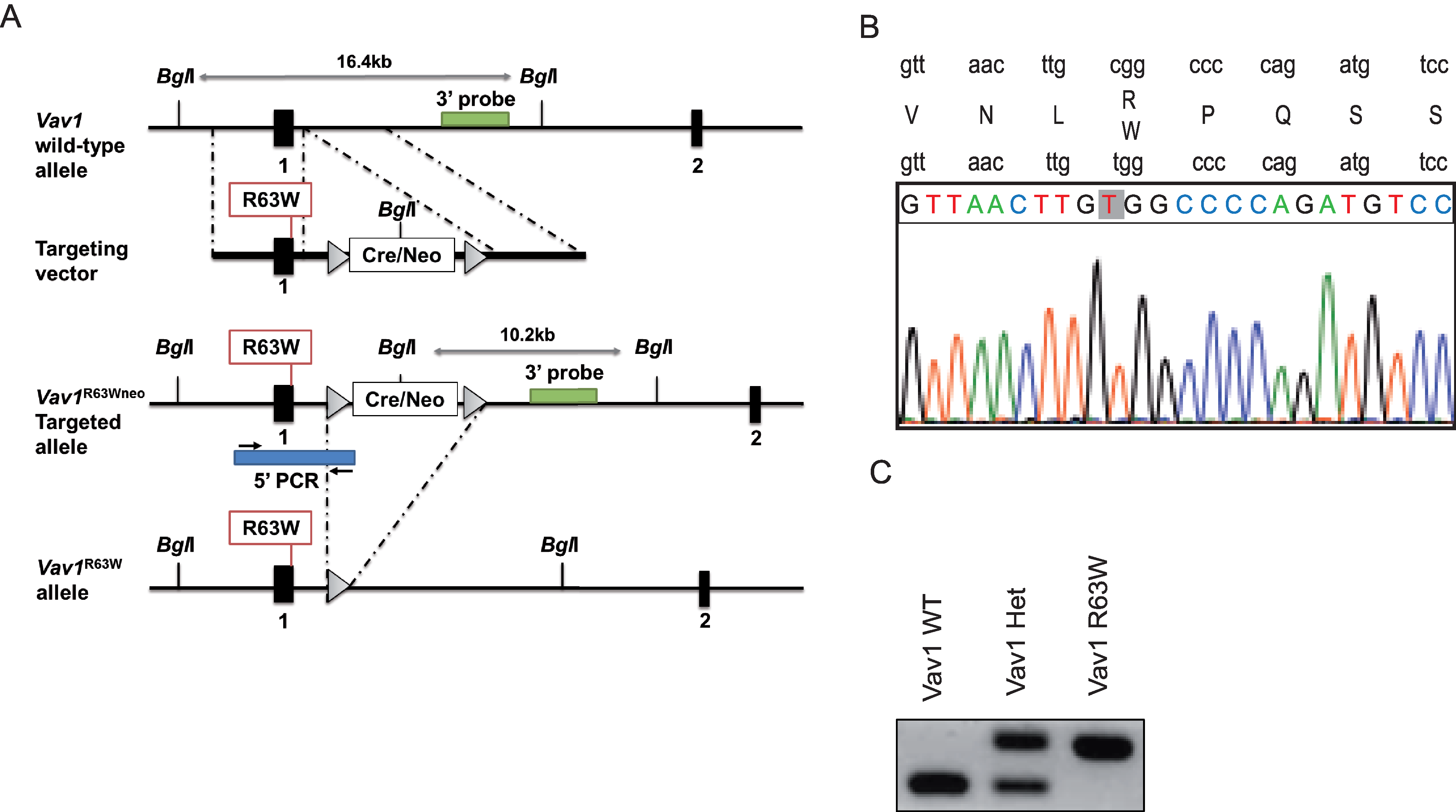

Supplement: S1 Fig — (A) Schematic drawing of the wild-type Vav1 allele before targeting, the targeting vector, the Vav1R63Wneo targeted allele generated in embryonic stem (ES) cells and the Vav1R63W allele with a single LoxP site after deletion of the Cre/Neo selection cassette following germline transmission. The targeting vector contains sequences encompassing exon 1 of the Vav1 gene. A mutation giving rise to the intended R63W substitution was introduced in exon 1 and a Cre-Neo selection cassette flanked by two LoxP sites was introduced in intron 1. Introduction of the neo gene into intron 1 was identified by Southern blotting of genomic DNA cut by BglI and probed with a 3’probe in intron 1. Predicted size of hybridizing bands are shown. The occurrence of an appropriate homologous recombination event at the 5’ side was screened by PCR using the following oligonucleotides: 5’-AAACCTAGTGGGCGCTCTCCA-3’ and 5’-TGACGAGTTCTTCTGAGCGG-3’. Black boxes represent exon 1 and 2 of Vav1, grey triangles represent LoxP sites, green box shows the location of the 3’ single-copy probe and blue box that of the PCR amplicon allowing to probe for proper recombination events at the 5’ end. ES clones containing the R63W allele were injected into FVB blastocysts to generate chimeric mice. Successful germline transmission was confirmed by sequencing (B) and PCR (C) with 5’-TGTAGGGGGCATCTGTCTGTCTG-3’ and 5’-AAATACCCTGGAGACTGCAGCAG-3’. This pair of primers amplifies a 203 bp band in the case of the wild-type allele and a 269 bp band in the case of the Vav1R63W allele. (TIF) [file pgen.1006185.s001.tif]

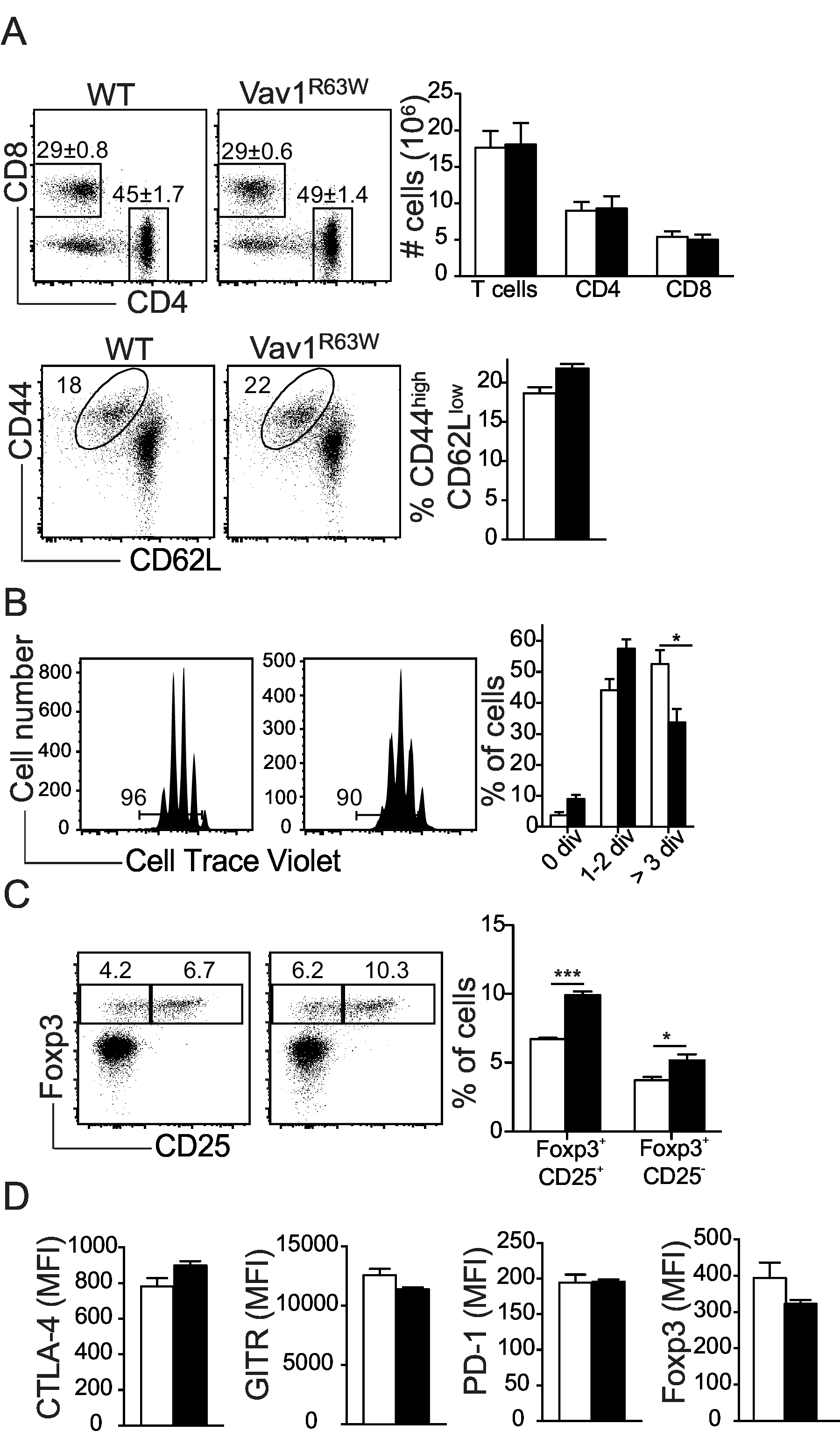

Supplement: S2 Fig — (A, upper panels) Representative dot plots of CD4 and CD8 T cells in the spleen of WT (n = 10) and Vav1R63W (n = 8) mice. The values on each cytometry profile represent the mean percentages of each population (mean ± SEM). Graphs show absolute numbers of each indicated population. (A, lower panels) Representative flow cytometry dot plots showing CD44 and CD62L expression on CD4 T cells in the spleen of WT and Vav1R63W mice. Graphs show the mean percentages of activated CD4+CD62LlowCD44high population. (B) Naïve CD4+CD62Lhigh T cells were purified from WT (n = 5) and Vav1R63W (n = 5) mice, stained with cell trace violet and stimulated with anti-CD3 and anti-CD28 antibodies for 72h. Proliferation of CD4 T cells was then analyzed by flow cytometry. Histograms represent the percentage of proliferating CD4 T cells. Graphs represent the percentage of non divided cells, cells divided one or two times and cells divided more than 3 times for the indicated genotypes. (C) Representative flow cytometry profiles of Foxp3+ T cells gated on CD4+ T cells in the spleen of WT (n = 10) and Vav1R63W (n = 8) mice. Graphs show mean percentages of CD4+Foxp3+CD25+ and CD4+Foxp3+CD25- T cells in the spleen. (D) Graphs represent the expression of characteristic markers by CD4+Foxp3+ T cells in the spleen of WT (n = 5) and Vav1R63W (n = 5) mice. ■: Vav1R63W mice; □: WT mice; *p≤0.05; ***p≤0.001. (TIF) [file pgen.1006185.s002.tif]

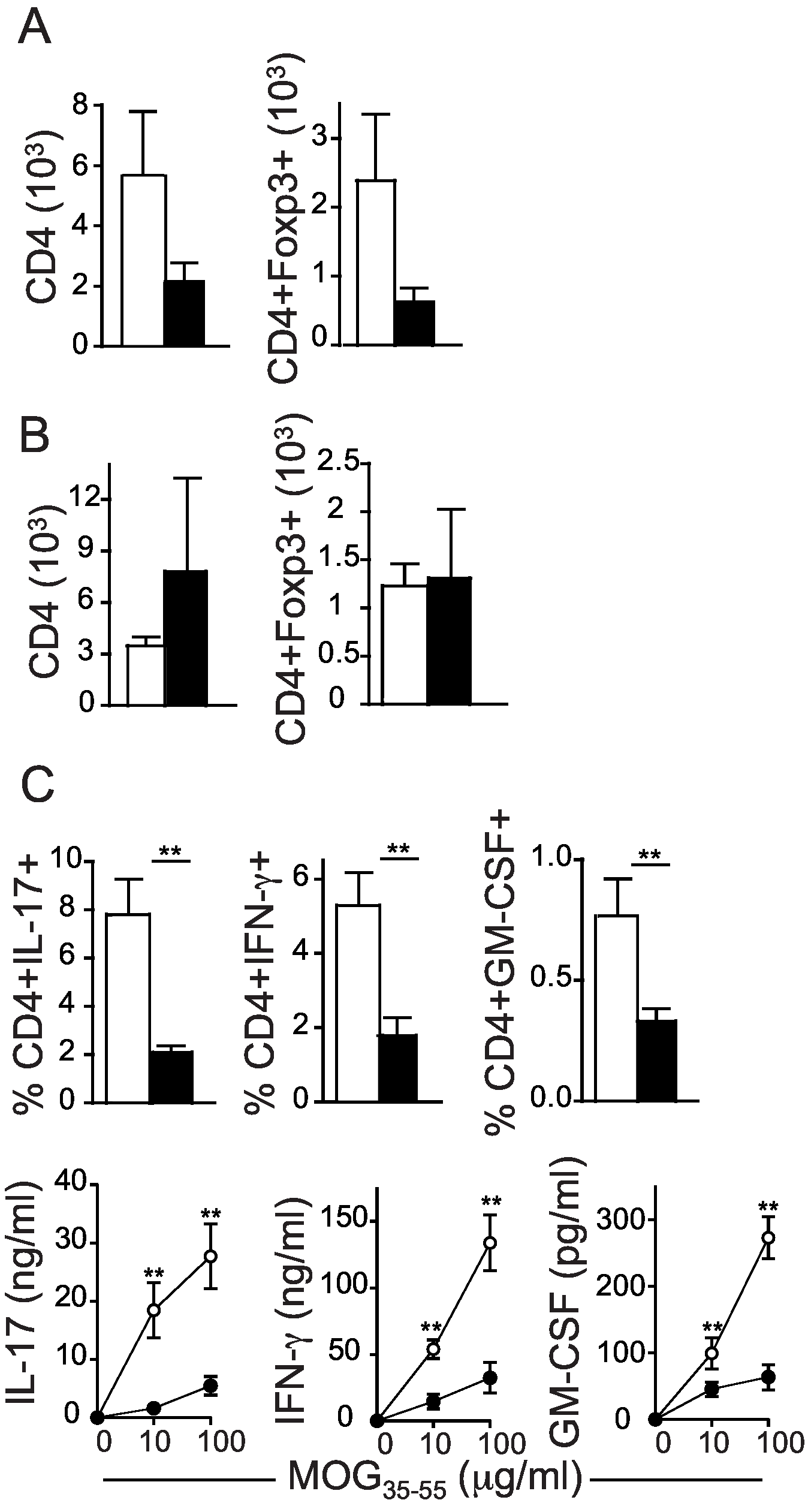

Supplement: S3 Fig — At day 30 after immunization, mononuclear cells were isolated from the CNS of individual mice (n = 7 per group). Graphs show the mean absolute numbers of CD4 T cells and CD4 Foxp3 Treg cells in the brain (A) and spinal cord (B). (C) Total LN cells collected on day 30 after immunization were re-stimulated for 72 hours with MOG35-55 peptide, graphs of the upper panels show cytokine expression by CD4+CD44high cells using intracellular staining after stimulation with 10 μg of MOG35-55. Lower panel show cytokine concentrations (IL-17, IFN-γ and GM-CSF) in the supernatants after stimulation with MOG35-55 peptide (10 or 100 μg). ■: Vav1R63W mice; □: WT mice; **p≤0.01 (TIF) [file pgen.1006185.s003.tif]
